# Influences of Realgar-*Indigo naturalis*, A Traditional Chinese Medicine Formula, on the Main CYP450 Activities in Rats Using a Cocktail Method

**DOI:** 10.1155/2017/2374624

**Published:** 2017-03-21

**Authors:** Huan-Hua Xu, Fei-Ran Hao, Mei-Xi Wang, Si-Jia Ren, Ming Li, Hong-Ling Tan, Yu-Guang Wang, Xiang-Lin Tang, Cheng-Rong Xiao, Qian-De Liang, Yue Gao, Zeng-Chun Ma

**Affiliations:** ^1^Guangdong Pharmaceutical University, Guangzhou 510006, China; ^2^Institute of Radiation Medicine, AMMS, Beijing 100850, China

## Abstract

The purpose of this work was to study the influences of Realgar-*Indigo naturalis* (RIF) and its principal element realgar on 4 main cytochrome P450 enzymes activities in rats. A simple and efficient cocktail method was developed to detect the four probe drugs simultaneously. In this study, Wistar rats were administered intragastric RIF and realgar for 14 days; mixed probe drugs were injected into rats by caudal vein. Through analyzing the pharmacokinetic parameter of mixed probe drugs in rats, we can calculate the CYPs activities. The results showed that RIF could inhibit CYP1A2 enzyme activity and induce CYP2C11 enzyme activity significantly. Interestingly, in realgar high dosage group, CYP3A1/2 enzyme activity was inhibited significantly, and different dosage of realgar manifested a good dose-dependent manner. The RIF results indicated that drug coadministrated with RIF may need to be paid attention in relation to drug-drug interactions (DDIs). Realgar, a toxic traditional Chinese medicine (TCM), does have curative effect on acute promyelocytic leukemia (APL). Its toxicity studies should be focused on. We found that, in realgar high dosage group, CYP3A1/2 enzymes activity was inhibited. This phenomenon may explain its potential toxicity mechanism.

## 1. Introduction

The complexity of medicine suggests that treatment protocols should be carefully designed, and construction of a prescription is an art in fighting disease. Increasing evidences demonstrate that, in treating diseases, especially cancer [1] and chronic diseases [2], therapeutic regimens containing multiple drugs with distinct but related mechanisms can usually amplify the therapeutic efficacies or reduce adverse effect of each agent, leading to maximal therapeutic efficacy with minimal adverse effects [3]. Traditional Chinese medicine (TCM) formula [4] is a unique medical system assisting the ancient Chinese in dealing with disease for thousands of years. Typically, formula consists of two or more types of medicinal herbs, animals, or minerals. In a formula, there is a component called “emperor” medicine which represents the principal component and exerts the main therapeutic effect, while other medicines serve as adjuvant ones to assist the therapeutic effect or reduce the adverse effect of the “emperor” medicine. As a matter of fact, at least in some formula, multiple components could hit multiple targets and exert synergistic therapeutic efficacies [5, 6]. However, in most formulas, essential compounds and precise mechanisms remain undefined, especially the formulas containing toxic elements, such as arsenic and mercury. In western countries, undefined mechanisms and excess toxic substance are the reasons to ban TCM, thus impeding the modernization of TCM.

Acute promyelocytic leukemia (APL) is characterized by the accumulation of immature promyelocytes in bone marrow (BM) and the presence of a specific chromosome translocation t(15:17)(q22:q21) in a vast majority of patients [7]. Interestingly, TCM doctor in China first used arsenic trioxide (ATO) to treated APL and this proved to be very effective [8, 9]. In 1997, 2 papers [10, 11] from investigators in Shanghai and Harbin, China, were published in* Blood Journal*, describing the mechanism of action and clinical results of treating APL patients with ATO. At the time, little was known about ATO treatment of leukemia patients in western medicine, and these 2 papers helped accelerate the development of ATO as a leukemia drug. Today, ATO has become the first choice in APL therapy [12]. Realgar-*Indigo naturalis *formula (RIF), which was invented by Huang [13], a TCM doctor in China, in the 1980s, is entirely based on TCM theories. Some most recent multicenter clinical trials showed that a complete remission rate of 96.08% (within 60 days) to 100% (about 107 days) was achieved in APL patients receiving RIF, with moderate adverse effects such as gastrointestinal discomfort and rash [14]. The “emperor” medicine of RIF is realgar (As_4_S_4_), while* Indigo naturalis, Salvia miltiorrhiza*, and* Radix pseudostellariae *serve as adjuvant. Despite the promising clinical studies and excellent curative effect in APL treatment, arsenic is well known for its toxic effects. RIF as an arsenic-containing preparation also raises public concern. Similar to realgar, many traditional medicines are forbidden in the US and European market because the p content of arsenic is higher than their tolerance limits [15, 16]. More in-depth studies of arsenic toxicology are very necessary to make those excellent drugs achieve their market recognition. Previous literature investigation showed that most studies of RIF focused on the molecular mechanism and clinical pharmacological effects. However the interactions between RIF and cytochrome P450 enzymes were not mentioned among these researches.

Cytochrome P450 enzymes (CYPs) is responsible for the metabolism of a variety of drugs, xenobiotics and endogenous substances. Over 90% of the clinical drugs are metabolized by human CYPs [17]. Among many CYPs isoforms, human CYP1A2, CYP2C9, CYP2E1, and CYP3A4 are involved in approximately 80% of the CYP-mediated drug metabolism clinically [18]. In this work, we chose rats as the experimental subjects. Although there exist differences in the category of subtypes, expression and catalytic activity of cytochrome P450s between human and rat, animal models are often used in the prediction of drugs and new ingredients metabolisms in preclinical study. In general, rat CYPs CYP1A2, CYP2C11, CYP2E1, and CYP3A1/2 have homologs in human CYPs. Moreover, the four rat CYP subtypes play important roles in the processes of attenuation and activation of exogenous substances in rodents. Hence, the results from rats are also useful for predicting the potential impact on the human. Induction or inhibition of the CYP activities has been recognized as the most important contributor to the unexpected or even serious clinical drug-drug interactions (DDIs) in clinic. Meanwhile, based on cytochrome P450 enzymes, system of TCM and its active ingredients or components synergy and toxicity reducing effect of combinations research achieved outstanding results in recent years. Wang et al. [19] and Zhang et al. [20] have found that PXR-mediated effects on CYP3A and CYP7A may contribute to the hepatoprotective property of glycyrrhizin and Tanshinone IIA against LCA-induced liver injury. Based on the hypothesis whether the RIF has similar effects on CYP enzymes, we think it is necessary to study the effect of RIF and its principle component (realgar) on CYPs.

This paper aims at elucidating the potential influences of RIF and realgar on the activities of four CYP450 isozymes (CYP1A2, CYP2C11, CYP2E1, and CYP3A1/2) in rats. A simplified and specific HPLC method was developed and validated to simultaneously quantify the four substrates in rat plasma as a cocktail. The changes of relative enzyme activity were evaluated as well.

## 2. Methods and Materials

### 2.1. Chemicals and Reagents

Realgar-*Indigo naturalis* formula tablets (Product number 150402) were obtained from Tiankang Pharmaceutical Co., Ltd. (Anhui, China). Realgar was purchased from Huamiao TCM Engineering Technology Center (Beijing, China). Caffeine (Product number 150439) and midazolam (Product number 150402) were purchased from National Institutes for Food and Drug Control (Beijing, China). Tolbutamide (Product number 1002039664) and chlorzoxazone (Product number 1001433956) were purchased from Sigma-Aldrich Co., Ltd. (Darmstadt, Germany). Diazepam (Product number 1503141) as internal standard (IS) was obtained from Kingyork Group Co., Ltd. (Tianjin, China). HPLC-grade acetonitrile was supplied by Fisher Scientific Company Inc. (Boston, USA). All other reagents were of analytical grade and purchased from Sinopharm Chemical Reagent Co., Ltd. (Beijing, China).

### 2.2. Animals

30 Sprague-Dawley rats (male, 180–200 g) were purchased from the animal experiment center of Academy of Military Medical Sciences of people's liberation army (Production license: SCXK(army)-2012-0004). Animal welfare and experimental procedures were strictly in accordance with the Guide for the Care and Use of Laboratory Animals (US National Research Council, 1996). All animals were kept in an environmentally controlled breeding room (temperature: 22 ± 2°C, humidity: 50 ± 5%, and 12 h dark/light cycle) for at least one week before starting the experiments and fed with standard laboratory food and water. Prior to each experiment, all of the rats were fasted for 12 h with free access to water.

### 2.3. HPLC of Biosamples

Chromatographic analysis was performed on a Waters 2695 Series (Waters Technologies, USA) LC system containing a quaternary pump, an online degasser, an autosampler, and a thermostatic column compartment set at 30°C. Chromatographic separation was conducted on Waters XSelect HSS T3 C18 column (4.6 mm × 250 mm, 5 *μ*m). The mobile phase consisted of a mixture of acetonitrile (A) and 50 mM phosphate buffer (pH 3.0) (B). Gradient elution was performed as follows: (1) mobile phase A was at 60% at 0 min, (2) an isocratic elution was maintained at 60% A from 0 min to 9 min, (3) a linear gradient was increased to 75% A from 9 min to 13 min, and (4) mobile phase A maintained at 75% from 13 min to 15 min. The flow rate was 1.0 ml/min. UV detection wavelength was 230 nm.

### 2.4. Biosamples Pretreatment

In our study, a conventional liquid-liquid extraction method was used to prepare the plasma sample [21]. After plasma samples thawed at room temperature, 150 *μ*l of plasma was mixed with 20 *μ*l internal standard (diazepam). The mixture was vortexed for 1 min. 600 *μ*l ethylacetate was added to centrifuge tube (1.5 ml). The mixture was vortex-mixed and centrifuged at 12000 ×g for 10 min to precipitate proteins. The organic layer was evaporated to dryness under a gentle stream of nitrogen at 40°C. Finally, the residue was dissolved in 200 *μ*l of the mobile phase and centrifuged at 12000 ×g for 10 min. 20 *μ*l of the supernatant was injected into HPLC system for analysis.

### 2.5. Method Validation

#### 2.5.1. Selectivity and Specificity

To investigate whether endogenous substances would interfere with the assay, blank plasma, blank plasma extracted by ethylacetate containing IS, blank plasma spiked with analytes, and a plasma sample collected at 45 min after administration intravenously (by caudal vein) of mixed probe drugs to rats were processed according to the procedures described above and analyzed.

#### 2.5.2. Linearity and LLOQ

The calibration curves of HPLC method were evaluated by analyzing a series of standard plasma samples at concentration from 0.2 to 200 *μ*g/ml for caffeine and from 0.4 to 400 *μ*g/ml for tolbutamide, chlorzoxazone, and midazolam. Using weighted least squares linear regression of the peak area of each probe drug, we can obtain the corresponding concentration (*C*). The lower limits of quantification (LLOQ) were defined as the plasma concentration giving a signal to noise ratio (*S*/*N*) > 10.

#### 2.5.3. Precision and Accuracy

Intraday/interday accuracies and precisions of the method were evaluated by analyzing the QC samples at 3 concentration levels in 6 replicates during a single day and in duplicate over 3 consecutive days. The precision was represented by relative standard deviation (RSD), which should be not be more than 15%. The accuracies were determined by calculating the percentage of measured concentrations to the nominal concentrations of each probe drug in QC samples and should be within 85–115%.

#### 2.5.4. Extraction Recovery

The extraction recovery was determined by comparing the response of analytes in postextract QC samples to that obtained from preextraction spiked samples. Experiments were performed at 3 concentration levels in 6 replicates. The results of extraction recovery should be stable and repeatable.

#### 2.5.5. Stability

The stability experiments of analytes in biosamples were investigated under a variety of storage and process conditions. Sample freeze-thaw stability was determined after 3 freeze-thaw cycles by comparing the concentrations with their nominal concentrations. For room temperature stability, the QC samples were prepared in the reconstituted solution for 24 h at room temperature and analyzed along with the freshly prepared set of QC samples. Samples were considered stable if the accuracies of samples were within 15% at different levels, and the precisions should not exceed 15%.

### 2.6. In Vivo Assay

#### 2.6.1. Drug Administration and Sampling

30 male Sprague-Dawley rats were randomly divided into 5 groups: control group, RIF group, and realgar group (low, middle, and high). Each group contained 6 rats. All drugs were administered orally. According to the dispensatory of Realgar-*Indigo naturalis* formula tablets and considering the conversion coefficient between rat and human, we have calculated the clinic equivalent dosage of RIF to be 720 mg/kg. In the RIF tablet, realgar accounts for 14.5% of the weight; based on the ratio, we selected the dose of different realgar groups, that is, 104.4, 1044, and 2088 mg/kg, and the 104.4 mg/kg was equivalent to the actual clinic dosage. Respectively, for consecutive 14 days, during this period, control group rats were administered oral normal pure water. In the 15th day, the mixed probe drugs containing caffeine, chlorzoxazone, tolbutamide, and midazolam were administered intravenously (by caudal vein) at a dosage of 2.5, 5, 5, and 5 mg/kg. Approximately 0.50 ml of blood samples was collected in heparinized tubes following the specific schedule: 0.17, 0.33, 0.5, 0.75, 1, 1.5, 2, 4, 8, 12, and 24 h. Each blood sample was immediately centrifuged for 15 min at 5000 rpm. The obtained plasma layers were separated and stored in microcentrifuge tubes at −20°C before analysis was performed as the procedure in Section 2.4.

### 2.7. Statistical Analysis

The data was generated and analyzed by Waters Empower Acquisition software (Waters Technologies). The pharmacokinetic parameters were analyzed by DAS pharmacokinetic program (Version 2.0; Chinese Pharmacological Society). All data were presented as mean ± SD One-way ANOVA test was employed to determine the differences of pharmacokinetic parameters by SAS statistical software. For all analyses, *p* < 0.05 was considered statistically significant; *p* < 0.01 was considered as extremely statistically significant. GraphPad prism software (Version 5.0) was used to draw statistical figures.

## 3. Results

### 3.1. Method Validation

#### 3.1.1. Selectivity and Specificity

**Figure 1:**
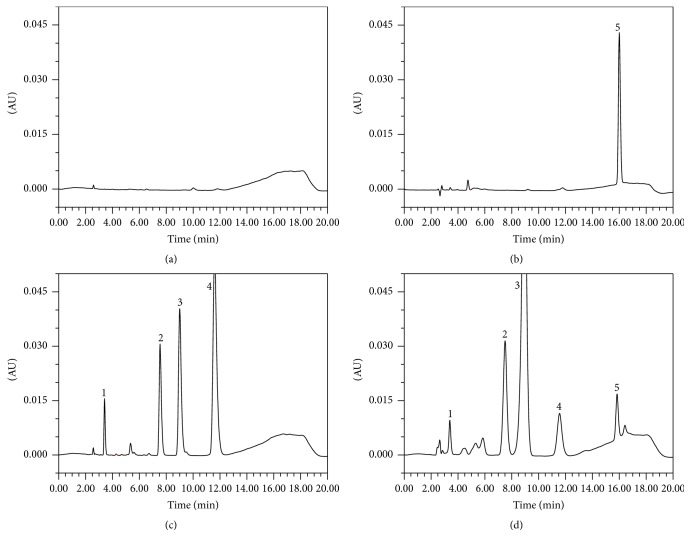
Representative chromatograms of blank plasma (a), blank plasma with IS (b), blank plasma with mixed probe drugs (c), and plasma sample collected at 45 min after oral administration of mixed probe drugs to rats (d). 1 = caffeine, 2 = chlorzoxazone, 3 = tolbutamide, 4 = midazolam, 5 = internal standard (IS) diazepam.

The retention time of relevant analytes was 3.41, 7.54, 9.02, and 11.58, respectively. No endogenous interfering peaks were observed at or near the retention time of analytes by comparing the chromatograms. Typical chromatograms of blank plasma, blank plasma extracted by ethylacetate containing IS, blank plasma spiked with analytes, and a plasma sample collected at 45 min after administration of mixed probe drugs to rats were shown in Figure 1. Meanwhile, the chromatograms of blank plasma showed that endogenous substances have no influence on the detection of analytes.

#### 3.1.2. Linearity and LLOQ

The standard curve, correlation coefficient of each analyte in the selected concentration range, and LLOQ were shown in Table 1. The calibrations were linear over a certain range in all biosamples with a correlation coefficient (*R*^2^) larger than 0.990.

#### 3.1.3. Precision and Accuracy

The obtained intraday/interday accuracy and precision data were summarized in Table 2. The intraday accuracies ranged from 87.90% to 104.60%, along with precisions ranging from 0.14% to 1.00%. Meanwhile, interday accuracies ranged from 87.64% to 104.48%, while precisions varied from 0.57% to 3.70%. The data suggested that the method was accurate and reproducible for simultaneous quantification of analytes in rat plasma.

#### 3.1.4. Extraction Recovery

As shown in Table 3, the extraction rate for caffeine, chlorzoxazone, tolbutamide, and midazolam was ideal which ranged from 85.65% to 99.18%.

#### 3.1.5. Stability

A summary of stability of each analyte was given in Table 4. The results indicated that the analytes in biosamples were of good stability.

### 3.2. Effects of RIF and Realgar on the CYP450 Activities in Rats

#### 3.2.1. Effects of RIF and Realgar on the Activity of CYP1A2 in Rats

**Figure 2:**
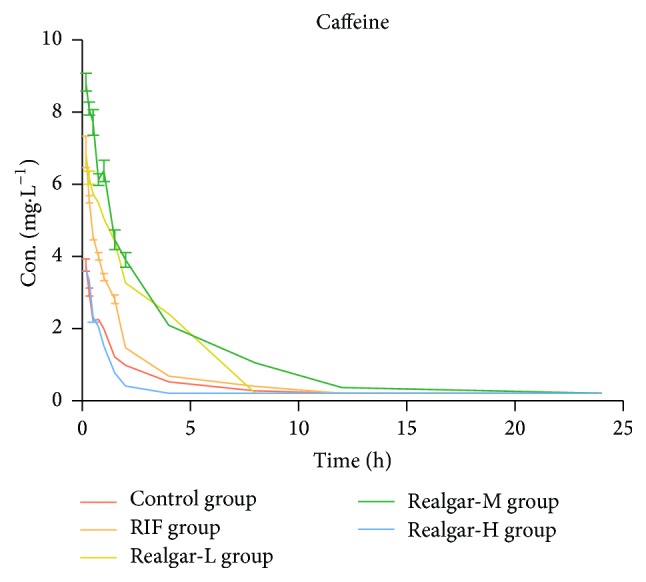
Plasma concentration-time curves of caffeine in rats after administered realgar and RIF for 14 days.

By comparing the pharmacokinetic parameter of caffeine in each group, we can calculate the CYP1A2 activity. As shown in Table 5 and Figure 2, compared with the control group, the AUC and *C*_max_ in RIF group increased and CL_*z*_ decreased. Both realgar low and middle dosage groups presented the same tendency as that of RIF group. However the realgar high dosage group showed the opposite tendency. Therefore, the pharmacokinetic behavior of caffeine indicated that RIF and realgar at low and middle dosage can inhibit the CYP1A2 activity, but high dosage realgar might induce the CYP1A2 activity.

#### 3.2.2. Effects of RIF and Realgar on the activity of CYP2E1 in Rats

**Figure 3:**
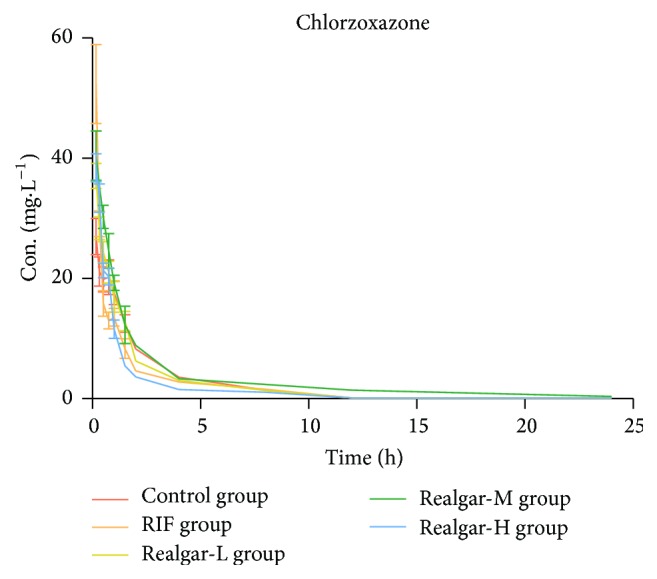
Plasma concentration-time curves of chlorzoxazone in rats after administered realgar and RIF for 14 days.

By comparing the pharmacokinetic parameter of chlorzoxazone in each group, we can calculate the CYP2E1 activity. As shown in Table 6 and Figure 3, compared with the control group, RIF and realgar low dosage group only significantly increased *C*_max_. This maybe indicated that RIF and low dosage realgar had no significant influence on CYP2E1 activity. In realgar middle group, AUC was increased, *t*_1/2_ was prolonged, and CL_*z*_ was decreased significantly; however, in realgar high dosage group, the tendency was the opposite. Therefore, the pharmacokinetic behavior of chlorzoxazone indicated that, in the realgar middle dosage group, the CYP2E1 activity was inhibited, but high dosage realgar might induce the CYP2E1 activity. The correlation between CYP2E1 activity and realgar dosage was nonlinear.

#### 3.2.3. Effects of RIF and Realgar on the Activity of CYP2C11 in Rats

**Figure 4:**
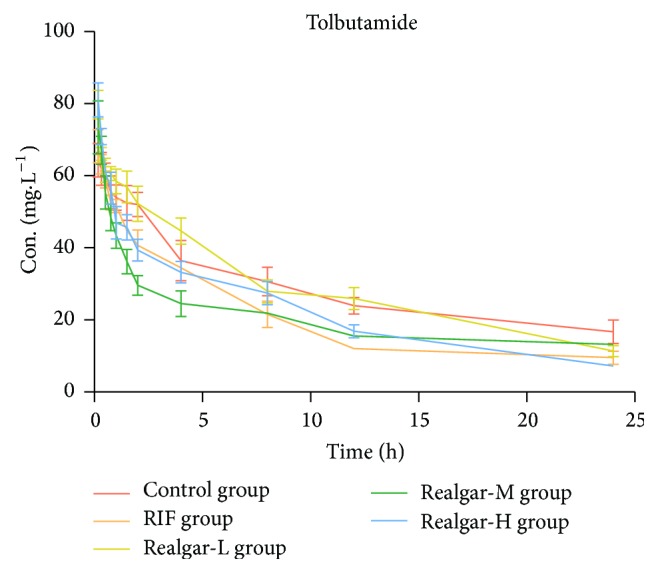
Plasma concentration-time curves of tolbutamide in rats after administered realgar and RIF for 14 days.

By comparing the pharmacokinetic parameter of tolbutamide in each group, we can calculate the CYP2C11 activity. As shown in Table 7 and Figure 4, compared with the control group, RIF group significantly decreased AUC and *t*_1/2_, and realgar middle and high groups showed the same tendency. The pharmacokinetic parameter of realgar low dosage group had no obvious difference compared with the control group. Therefore, the pharmacokinetic behavior of tolbutamide indicated that realgar had no significant influence on CYP2C11 activity at low dosage, but it might induce the CYP2C11 activity at middle and high dosage. The correlation between CYP2C11 and realgar dosage was nonlinear. This phenomenon may need further investigation.

#### 3.2.4. Effects of RIF and Realgar on the Activity of CYP3A1/2 in Rats

**Figure 5:**
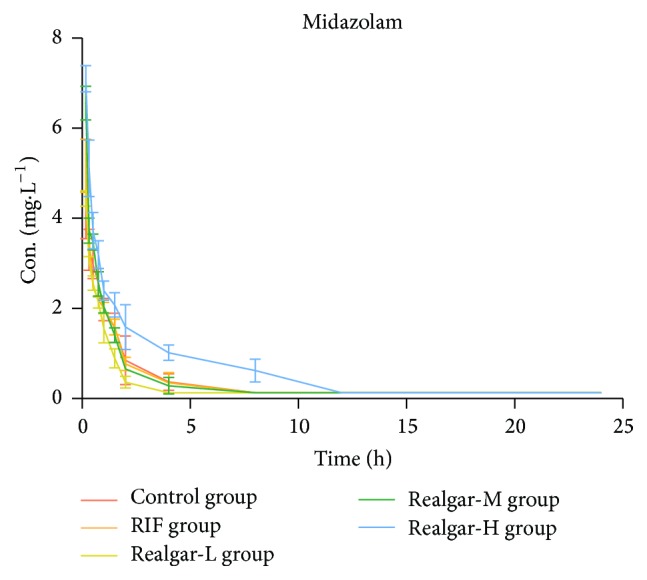
Plasma concentration-time curves of midazolam in rats after administered realgar and RIF for 14 days.

By comparing the pharmacokinetic parameter of midazolam in each group, we can calculate the CYP3A1/2 activity. As shown in Table 8 and Figure 5, compared with the control group, the pharmacokinetic parameters in RIF group had no significant change. In realgar high dosage group, AUC and *C*_max_ were significantly increased; *t*_1/2_ was prolonged, while CL_*z*_ had no obvious change. The realgar at different dosage manifested a good dose-dependent manner. Therefore, the pharmacokinetic behavior of midazolam indicated that RIF and realgar at low and middle dosage had no significant influence on CYP3A1/2 activity, but high dosage realgar might inhibit the CYP3A1/2 activity.

## 4. Discussion

In this work, we first established a simple and useful method for probe drugs quantitative analysis and then verified it. Compared with the method proposed by Yang et al. [22], our method was more time-saving and has simpler mobile phase composition. We adjusted the mobile phase pH to 3 to get more symmetrical peaks. Samples had shorter retention time as well. Although we only detected 4 kinds of probe drugs simultaneously, we could extend the method to more drugs. The results showed that, by adjusting the mobile phase, we could also detect other 4 commonly used probe drugs (i.e., omeprazole, metoprolol, phenacetin, and dextromethorphan), which were recommended by FDA in a single run. Based on this method, researchers are free to detect all combinations of the above 8 probe drugs according to their demands. The retention time can also be adjusted through adjusting the mobile phase. Extension method and the corresponding data were not shown in this article.

Through evaluating the influence of RIFs on the activity of 4 CYP subtypes, we are able to predict and improve the effectiveness and reliability of RIF on clinical applications. The results showed that RIF of clinical dosage did not alter the CYP3A1/2 or CYP2E1 activity but inhibited the CYP1A2 activity and induced the CYP2C11 activity. The influence of RIF on the activity of the 4 enzymes can be used to predict drug-drug interactions and provide guidance for RIF's combination with other drugs.

Another purpose of this study is to evaluate the influence of different doses of realgar on the 4 CYP enzymes. The research by Aihua et al. [23] showed that the long-term use of realgar at 8 and 16 times of the clinical dose may cause poisonous effects to kidney and liver and impair blood system. Lu et al. [24] also recommended 10 times of the clinical dose as a proper dosage to study realgar. In order to avoid severe adverse effects during experimental period and cause mild damage to rats, we set 20 times of the clinical dose as the dosage of high dosage realgar group based on the results from preliminary experiments. The results showed that CYP1A2 activity was inhibited in realgar low and middle dosage groups, but the activity was induced in high dosage group. As to CYP2E1, its activity was not affected in low dosage group, inhibited in middle dosage group, and induced in high dosage group. For CYP2C11, its activity was not affected in low dosage group, but it was induced in middle and high dosage groups. For both CYP2E1 and CYP2C11, there was no dose-effect relationship. Interestingly, realgar of different dosages manifested a good dose-dependent manner on CYP3A1/2. With the increase of realgar dose, the CYP3A1/2 enzyme activity significantly decreased. CYP3A subtype is the most important subtype of CYP450 enzymes in drug metabolism, which involves 60% of drug metabolism [25]. In the previous studies (data unpublished), mild liver cell degeneration and ALT/AST biochemical indicators change was observed in realgar high group. This may be related to the accumulation of realgar. Whether the accumulated toxicant was associated with the inhibition of the activity of CYP3A enzymes needs further study.

Increasing evidences demonstrate that research on CYP450 enzymes not only can be used to predict the DDIs, but also can be applied in the study of the characteristics of TCM compatibility. Research results based on CYP450 enzymes can reveal the scientific connotation of classical TCM formula compatibility rationality, especially in the study of the attenuated toxic effects of TCM formula. Research on CYP450 enzymes has become a tool and plays a more and more important role in TCM synergism and toxicity reducing research. In the future study, we are looking forward to exploring the relationship between the toxicity of accumulated realgar and CYP3A enzyme activity.

## 5. Conclusion

RIF at clinical dosage can inhibit CYP1A2 enzyme activity and induce CYP2C11 enzyme activity significantly. In realgar high dosage group, CYP3A1/2 enzyme activity was inhibited significantly, and different dosage of realgar manifested a good dose-dependent manner.

## Figures and Tables

**Table 1:** Standard curves, correlation coefficients, linear ranges, and LLOQ of analytes in rat plasma.

Number	Standard curves	*R* ^2^	Linear range (*μ*g·ml^−1^)	LLOQ (*μ*g·ml^−1^)
Caffeine	*Y* = 9.60*e* + 003*X* − 7.23*e* + 003	0.999	0.21–215	0.21
Chlorzoxazone	*Y* = 1.71*e* + 004*X* − 2.56*e* + 004	0.999	0.41–406.4	0.11
Tolbutamide	*Y* = 2.78*e* + 004*X* − 4.11*e* + 004	0.999	0.44–445.2	0.09
Midazolam	*Y* = 5.12*e* + 004*X* − 8.16*e* + 004	0.999	0.42–440.1	0.13

**Table 2:** Intra-/interday accuracy and precision of the developed assay (*n* = 6).

Number	QC conc. (*μ*g·ml^−1^)	Intraday			Interday		
		Calc. conc. (*μ*g·ml^−1^)	RSD (%)	Accuracy (%)	Calc. conc. (*μ*g·ml^−1^)	RSD (%)	Accuracy (%)
Caffeine	5.38	5.32 ± 0.01	0.14	98.97 ± 0.14	5.32 ± 0.04	0.79	98.81 ± 0.78
	10.75	9.89 ± 0.03	0.30	92.02 ± 0.27	9.93 ± 0.07	0.66	92.38 ± 0.61
	51.80	47.76 ± 0.48	1.00	92.20 ± 0.92	46.91 ± 1.74	3.70	90.56 ± 3.35

Chlorzoxazone	10.16	10.63 ± 0.03	0.25	104.60 ± 0.26	10.62 ± 0.07	0.64	104.48 ± 0.67
	20.32	19.96 ± 0.03	0.17	98.21 ± 0.17	20.29 ± 0.43	2.11	99.85 ± 2.10
	101.60	96.35 ± 0.40	0.42	94.84 ± 0.40	96.72 ± 0.56	0.57	95.20 ± 0.55

Tolbutamide	11.13	10.71 ± 0.06	0.56	96.22 ± 0.54	10.59 ± 0.07	0.63	95.17 ± 0.60
	22.26	20.08 ± 0.05	0.23	90.22 ± 0.21	19.97 ± 0.25	1.23	89.69 ± 1.10
	109.60	96.34 ± 0.38	0.39	87.90 ± 0.34	96.05 ± 1.70	1.77	87.64 ± 1.55

Midazolam	11.01	10.64 ± 0.02	0.18	96.60 ± 0.18	10.58 ± 0.17	1.56	96.12 ± 1.50
	22.02	19.88 ± 0.04	0.20	90.28 ± 0.18	19.65 ± 0.18	0.92	89.24 ± 0.82
	105.30	96.37 ± 0.38	0.40	91.52 ± 037	94.66 ± 1.93	2.04	89.90 ± 1.83

**Table 3:** Recovery of the developed assay (*n* = 6).

Number	QC conc. (*μ*g·ml^−1^)	Recovery (Mean ± S.D.)	RSD (%)
Caffeine	5.38	96.71 ± 3.05	3.15
	10.75	91.04 ± 2.16	2.38
	51.80	93.64 ± 5.54	5.92

Chlorzoxazone	10.16	99.18 ± 2.47	2.49
	20.32	97.91 ± 3.41	3.49
	101.60	94.98 ± 2.00	2.11

Tolbutamide	11.13	90.04 ± 3.80	4.22
	22.26	90.81 ± 2.88	3.18
	109.60	85.65 ± 2.26	2.64

Midazolam	11.01	90.95 ± 4.25	4.67
	22.02	90.41 ± 1.95	2.16
	105.30	95.27 ± 9.10	9.55

**Table 4:** Stability of analytes in rat plasma (*n* = 6).

Number	QC conc. (*μ*g·ml^−1^)	Room temperature stability			Freeze-thaw stability		
		Calc. conc. (*μ*g·ml^−1^)	RSD (%)	Accuracy (%)	Calc. conc. (*μ*g·ml^−1^)	RSD (%)	Accuracy (%)
Caffeine	5.38	5.30 ± 0.05	0.90	98.54 ± 0.88	5.27 ± 0.08	1.44	97.95 ± 1.41
	10.75	9.74 ± 0.14	1.44	90.92 ± 1.3	9.87 ± 0.12	1.22	91.66 ± 1.09
	51.80	47.38 ± 0.89	1.88	91.30 ± 1.59	47.82 ± 1.07	2.23	92.24 ± 1.85

Chlorzoxazone	10.16	10.63 ± 0.09	0.86	104.66 ± 0.90	10.59 ± 0.08	0.79	104.28 ± 0.83
	20.32	20.20 ± 0.35	1.74	99.98 ± 2.05	20.40 ± 0.43	2.13	100.02 ± 2.10
	101.60	96.23 ± 0.68	0.70	94.67 ± 0.61	96.41 ± 0.48	0.49	94.81 ± 0.46

Tolbutamide	11.13	10.64 ± 0.17	1.62	95.57 ± 1.55	10.47 ± 0.29	2.74	94.06 ± 2.57
	22.26	20.32 ± 0.41	2.00	91.06 ± 1.71	20.03 ± 0.25	1.25	89.94 ± 1.01
	109.60	95.52 ± 2.17	2.27	87.27 ± 1.79	94.39 ± 1.29	1.37	86.30 ± 1.14

Midazolam	11.01	10.70 ± 0.29	2.71	97.21 ± 2.63	10.51 ± 0.13	1.22	95.45 ± 1.17
	22.02	19.90 ± 0.05	0.27	90.40 ± 024	19.78 ± 0.11	0.55	89.76 ± 0.49
	105.30	95.78 ± 0.55	0.57	91.12 ± 0.61	95.20 ± 2.52	2.65	90.28 ± 2.16

**Table 5:** Effect of RIF and realgar (different levels) on the pharmacokinetics of caffeine (*n* = 6).

Parameter/unit	Control	RIF	Realgar-L	Realgar-M	Realgar-H
AUC_(0–*t*)_/mg·L^−1^·h	10.83 ± 1.00	15.89 ± 0.81^*∗∗*^	24.58 ± 0.22^*∗∗*^	31.01 ± 2.46^*∗∗*^	8.46 ± 0.37^*∗∗*^
AUC_(0–*∞*)_/mg·L^−1^·h	11.80 ± 1.73	16.63 ± 1.69^*∗∗*^	26.00 ± 0.64^*∗∗*^	31.17 ± 2.60^*∗∗*^	9.36 ± 0.18^*∗∗*^
*t*_1/2*z*_/h	6.86 ± 3.00	3.85 ± 0.41	5.09 ± 1.32	3.07 ± 0.68^*∗∗*^	7.53 ± 1.48
CL_*z*_/L·h^−1^·kg^−1^	0.22 ± 0.03	0.15 ± 0.01^*∗∗*^	0.10 ± 0.01^*∗∗*^	0.08 ± 0.01^*∗∗*^	0.27 ± 0.01^*∗∗*^
*C*_max_/mg·L^−1^	3.80 ± 0.37	6.93 ± 1.03^*∗∗*^	6.85 ± 0.26^*∗∗*^	9.01 ± 0.36^*∗∗*^	3.66 ± 0.31

^*∗∗*^ *p* < 0.01 versus control group.

**Table 6:** Effect of RIF and realgar (different levels) on the pharmacokinetics of chlorzoxazone (*n* = 6).

Parameter/unit	Control	RIF	Realgar-L	Realgar-M	Realgar-H
AUC_(0–*t*)_/mg·L^−1^·h	60.88 ± 3.83	59.54 ± 1.79	61.80 ± 1.48	86.49 ± 6.03^*∗∗*^	47.96 ± 0.74^*∗∗*^
AUC_(0–*∞*)_/mg·L^−1^·h	60.99 ± 3.78	59.97 ± 1.69	62.01 ± 1.44	89.58 ± 6.83^*∗∗*^	48.36 ± 0.98^*∗∗*^
*t*_1/2*z*_/h	2.03 ± 1.05	3.34 ± 1.19	2.45 ± 1.16	5.76 ± 1.17^*∗∗*^	3.50 ± 1.62
CL_*z*_/L·h^−1^·kg^−1^	0.08 ± 0.01	0.08 ± 0.01	0.08 ± 0.01	0.06 ± 0.01^*∗∗*^	0.10 ± 0.00^*∗∗*^
*C*_max_/mg·L^−1^	27.33 ± 2.21	52.34 ± 6.57^*∗∗*^	37.07 ± 2.07^*∗∗*^	40.74 ± 3.61^*∗∗*^	38.78 ± 1.87^*∗∗*^

^*∗∗*^*p* < 0.01 versus control group.

**Table 7:** Effect of RIF and realgar (different levels) on the pharmacokinetics of tolbutamide (*n* = 6).

Parameter/unit	Control	RIF	Realgar-L	Realgar-M	Realgar-H
AUC_(0–*t*)_/mg·L^−1^·h	688.30 ± 61.38	489.08 ± 23.12^*∗∗*^	696.78 ± 34.11	490.21 ± 24.92^*∗∗*^	537.08 ± 30.88^*∗∗*^
AUC_(0–*∞*)_/mg·L^−1^·h	1016.61 ± 227.80	605.73 ± 27.78^*∗∗*^	870.12 ± 42.54	740.39 ± 161.75^*∗*^	629.69 ± 42.00^*∗∗*^
*t*_1/2*z*_/h	15.43 ± 6.39	9.63 ± 1.96	10.48 ± 1.25	15.57 ± 6.49	8.88 ± 1.59^*∗*^
CL_*z*_/L·h^−1^·kg^−1^	0.01 ± 0.01	0.01 ± 0.01	0.01 ± 0.00	0.01 ± 0.00	0.01 ± 0.00
*C*_max_/mg·L^−1^	65.01 ± 4.44	68.31 ± 4.52	79.74 ± 3.95^*∗*^	74.32 ± 6.99^*∗*^	81.07 ± 4.70^*∗∗*^

^*∗*^*p* < 0.05 versus control group, ^*∗∗*^*p* < 0.01 versus control group.

**Table 8:** Effect of RIF and realgar (different levels) on the pharmacokinetics of midazolam (*n* = 6).

Parameter/unit	Control	RIF	Realgar-L	Realgar-M	Realgar-H
AUC_(0–*t*)_/mg**·**L^−1^**·**h	8.92 ± 1.23	9.04 ± 0.87	6.33 ± 0.14^*∗∗*^	9.26 ± 0.54	15.73 ± 1.36^*∗∗*^
AUC_(0–*∞*)_/mg**·**L^−1^**·**h	31.38 ± 16.31	32.13 ± 16.99	22.05 ± 11.90	32.63 ± 17.46	37.50 ± 50.09^*∗*^
*t*_1/2*z*_/h	4.48 ± 1.57	4.39 ± 1.87	4.40 ± 1.20	4.81 ± 1.38	6.29 ± 1.92^*∗*^
CL_*z*_/L**·**h^−1^**·**kg^−1^	0.22 ± 0.16	0.23 ± 0.18	0.33 ± 0.25	0.22 ± 0.18	0.25 ± 0.10
*C*_max_/mg**·**L^−1^	4.08 ± 0.52	5.17 ± 0.90^*∗∗*^	4.44 ± 0.17	6.56 ± 0.38^*∗∗*^	7.10 ± 0.29^*∗∗*^

^*∗*^*p* < 0.05 versus control group, ^*∗∗*^*p* < 0.01 versus control group.
